# Translation and validation of the caregiving burden scale for family caregivers of children with cancer in chinese population

**DOI:** 10.1186/s12912-024-02204-4

**Published:** 2024-08-06

**Authors:** Xinyi Xu, Yating Yu, Li Tang, Qiurong Chen, Shuai Xie, Yao Cen, Xian Zhang, Lihua Min, Xiaorong Mao

**Affiliations:** 1 School of Medicine, University of Electronic Science and Technology of China, Chengdu, 610072 China; 2 Department of Nursing, The Quzhou Affiliated Hospital of Wenzhou Medical University, Quzhou People’s Hospital, Zhejiang, 324000 China; 3 Department of Pediatric Care Unit, Sichuan Provincial People’s Hospital, Chengdu, 610072 China; 4 Department of Vascular Surgery, Sichuan Provincial People’s Hospital, Chengdu, 610072 China; 5 Department of Pediatric Hematologic Oncology Nursing, West China Second University Hospital, Sichuan University/West China School of Nursing,Sichuan University, 2Key Laboratory of Birth Defects and Related Diseases of Women and Children (Sichuan University), Ministry of Education, Chengdu, 610041 China; 6 Department of Pediatrics, Mianyang Central Hospital, School of Medicine, University of Electronic Science and Technology of China, Mianyang, 621000 China; 7 Department of Pediatric Oncology, Sichuan Clinical Research Center for Cancer, Sichuan Cancer Hospital & Institute, Sichuan Cancer Center, Affiliated Cancer Hospital of University of Electronic Science and Technology of China, Chengdu, 610041 China; 8 Department of Pediatric Oncology, Chengdu Women’s and Children’s Central Hospital, School of Medicine, University of Electronic Science and Technology of China, Chengdu, 611731 China; 9 Department of Nursing, Sichuan Provincial People’s Hospital, School of Medicine, University of Electronic Science and Technology of China, Chengdu, 610072 China

**Keywords:** Caregiving burden, Children with cancer, Family caregivers, Reliability, Validity, China

## Abstract

**Background:**

Effective response and reducing the burden of family care for children with cancer is critical, and China currently lacks a specific assessment tool.

**Aims:**

This study aimed to translate and validate the Caregiving Burden Scale for Family Caregivers of Children with Cancer (CBSFC-CC) and then test and implement the tool.

**Methods:**

According to the Beaton cross-cultural debugging guide, preliminary Chinese version of CBSFC-CC scale was formed, which was suitable for Chinese language environment and clinical context. Exploratory factor analyses (EFA) and confirmatory factor analyses (CFA) were performed to verify structural validity. Convergent validity, discriminant validity and reliability were also conducted.

**Results:**

A total of 529 family caregivers of children with cancer participated in the survey. EFA extracts and combines four factors and explained 65.80% of the total variation. CFA proved that all the goodness-of-fit indicators were acceptable. The Cronbach’s alpha of the Chinese version of CBSFC-CC was .96, and the test–retest reliability coefficient was .95. Four dimensions and 29 items were identified in the final Chinese version of CBSFC-CC.

**Conclusion:**

The chinese version CBSFC-CC is scientifically reasonable and has good reliability and validity, which can be applied to the investigation of the nursing burden of family caregivers of children with cancer in China.

**Supplementary Information:**

The online version contains supplementary material available at 10.1186/s12912-024-02204-4.

## Background

Caregivers are those who provide extensive assistance to patients with chronic diseases or disabilities and provide the most care during their illness or disability, mainly classified as formal and informal. Formal caregivers are family doctors and trained carers or public service workers who are employed for a fee. The latter refers to relatives who provide free care for the family, including parents, grandparents, maternal grandparents, siblings, friends, or neighbors [1]. Family caregivers of children with cancer are often called as informal caregivers, but it can be extremely challenging for family caregivers who are not specially trained.

To date, the burden of care remains a complex concept with a multidimensional structure. Hoening and Hamilton [2] first put forward the concept of burden and divided it into subjective and objective burdens. Subjective burden relates to the caregiver's feelings about performing the caregiving function, while *objective* burden is defined as events or activities related to adverse caregiving experiences. Later, Zarit, Reever, and Bach-Peterson described burden as "the degree to which caregivers perceive the emotional, physical, social, and economic status of their relatives" [3]. Collins et al. also proposed that caregiver burden refers to other negative outcomes, such as psychological distress, physical health problems, economic and social stress, damaged family relationships, and feelings of hopelessness [4]. This study is based on the research of scholars at home and abroad on the concept of "caregiver burden" and finally adopts the concept definition proposed by Liu et al. [5] in 2020, that is, caregiver burden refers to the multifaceted pressure level endured by caregivers due to long-term care of family members, and such pressure level will lead to anxiety, burnout, depression, and other problems for caregivers.

The growing number of children with cancer places a considerable burden on tens of millions of family caregivers. Cancer will not only affect the growth and development of children, physical and mental health, but also affect the everyday life of family caregivers, resulting in the breakdown of regular family order, the relationship between children and siblings is involved, and the relationship between partners deteriorates [6]. Family caregivers are the people who have the most prolonged and intimate contact with their children daily. Children will be keenly aware their parents' the poor physical and mental state and then produce negative emotions, which significantly affects the degree of cooperation and treatment effect of children's clinical treatment. According to The first global report on the burden of childhood cancer published by The Lancet, China has the second highest burden of childhood cancer [7].

Using a reliable and valid scale is an effective method to assess the caregiving burden of family caregivers of children with cancer. Currently, the primary tools used to assess Caregiver Burden for family caregivers of children with cancer include Zarit Caregiver Burden Interview (ZBI) [8], Caregiver Burden Inventory (CBI) [9], Caregiver Burden Scale (CBS) [10], Parent Experience of Child Illness (PECI) [11], Caregiver Burden Scale (with positive entries)(CSI +) [12]. However, most of these tools have a long history of development. They primarily targeted caregivers of patients with chronic diseases such as Alzheimer's, Parkinson's, mental disorders, or inflammatory bowel disease [13–16] and lacked population specificity. Currently, there is no specific tool to measure the burden of family caregivers of children with cancer, which is not conducive to improving the fundamental understanding of the burden of family caregivers of children with cancer and can not provide targeted solutions and social support.

In 2021, Cevik Ozdemir et al. compiled the CBFSC-CC, which assessed the nursing burden of family caregivers of children with cancer from a multi-dimensional perspective, covering four dimensions of physical, emotional, psychological, socio-cultural and economic burden [17]. Compared with the ZBI, CBI, CBS, PECI, and CSI + scales, the CBFSC-CC contains more specific questions about the burden of family caregivers for children with cancer. The scale has only been tested for psychological characteristics by a Turkish development team [17], and the results show that it is a high-quality measurement tool. Based on the actual situation in China, the introduction of CBFSC-CC can not only provide a specific measurement tool for assessing the caregiver burden of Chinese children with cancer but also provide a valuable reference for further research on related intervention measures. Therefore, this study aims to sinicize CBSFC-CC and evaluate the validity and reliability of the Chinese version of CBSFC-CC.

## Methods

### Translation and cross-cultural adaptation

We got in touch with those who developed the CBSFC-CC, Dr. Hamide Nur Cevyk Ozdemyr, and got his authorization. The scale was sinicized by Beaton’s cross-cultural debugging guide [18]. After initial application and verification by the original author, three items were removed and 36 items remained for the final version of the CBSFC-CC [17]. Considering the possible cultural differences, after consulting with the Sinicization experts on the scale, this study decided to carry out Sinicization and cross-cultural debugging of all the initial items (39 items) and then adjust them according to the actual situation in China.

In the first step, CBSFC-CC was translated into Chinese by a clinical medical master and a nursing doctor proficient in English independently. Before the translation, they were introduced to the relevant definition and clinical status of the nursing burden of family caregivers of children with cancer. They were also informed that the translation process should pay attention to the semantic equivalence of the articles. At the end of the translation, the researchers and the two participants discussed the questions together to form an integrated version. Next, the integrated version was translated back into English by an English master and a finance master independently, both of whom had never been exposed to the original scale. In addition to translators and back-translators, the expert committee consists of a clinical medical expert engaged in pediatric hematology and tumor-related diseases treatment for more than 20 years, two nursing professors engaged in pediatric clinical care, teaching and nursing management for more than 30 years, and a professor working at a university and responsible for English teaching for more than 30 years.

After discussion by the expert committee, we randomly selected 30 family caregivers of children with cancer for pre-trial, all meeting this study's inclusion and exclusion criteria. After the completion of the scale, the researcher conducted a one-to-one interview to record the family caregivers' understanding of all items, whether there were words and sentences they did not understand, and how to express better help them understand the meaning of the items. Of these, 24 considered that there was no need for changes, and 6 made suggestions, mainly focusing on articles 23, 33, and 38, which were discussed with the expert committee and eventually revised and simplified the expression of some sentences. Finally, the report formed in the cross-cultural debugging process is sorted out and sent to the author of the original scale by email.

### Samples and setting

This is a cross-sectional study. From July to December2022, the convenient sampling method was used to investigate family caregivers of children with cancer hospitalized or followed up in multiple hospitals in Sichuan Province. The inclusion criteria: the participants were family caregivers of children with cancer aged 0 to 18 years, the care time was > 6 h/day, the duration was ≥ 3 months, and they have a good understanding and expression when communicating with others. The exclusion criteria are those with more serious physical, mental, psychological, or cognitive disorders and the staff paid to care for them.

All participants voluntarily participated in the study, knowing that this was a completely anonymous survey intended for academic purposes only and that there would be no harm or additional cost to them. According to Worthington's opinions [19], the sample size for exploratory factor analysis should be more than 300 cases, and the sample size for confirmatory factor analysis should be no less than 200 cases. In order to ensure the accuracy of this study, a total of 527 family caregivers of children with cancer were surveyed by questionnaire.

## Measures

### General information questionnaire

The general information questionnaire includes both family caregivers and children with cancer. The information of family caregivers mainly includes age, gender, ethnicity, marital status, education level, current working status, number of children, etc. The information of children with cancer includes the type of disease, time of diagnosis, current disease status, and whether there is metastasis.

### Chinese version of the CBSFC-CC

CBFSC-CC [17] is a 36-item scale with four dimensions of physical, emotional, psychological, sociocultural and economic burden. Each item was scored using a 5-point Likert scale, with a minimum of 36 and a maximum of 180. 36 ~ 72 was regarded as no burden or almost no burden; 73 ~ 108 was considered as mild and moderate burden; 109–144 was viewed as moderate or severe burden; Between 145 and 180 is classified as extremely heavy. This study included 39 entries from the original CBSFC-CC, with values ranging from 1(never) to 5(always). The lowest score is 39 and the highest is 195. The higher the score, the heavier the level of care burden.

### Data collection

Due to the impact of COVID-19, this study adopts the form of electronic questionnaire survey. Before the questionnaire was sent to the potential participants, We convened a meeting for the investigator to give consistent explanations during the survey. At the same time, in the first part of the questionnaire, we briefly introduced the background, purpose, and significance of this study to family caregivers of children with cancer and conveyed the principle of informed consent to them. Instructions are set to help family caregivers better grasp and understand the entries. After completing the questionnaire, the prominent researcher conducted a preliminary review, and deleted the sample data that were lower than the lowest score (39 points). More than 1/2 items were not answered, the response time was less than 5 min, and they were invalid. Participants will then receive a small thank-you gift.

### Statistical analyses

SPSS26.0 was used to describe the general information of family caregivers of children with cancer through indicators such as frequency and percentage. The determination value and correlation coefficient method were used to test the differentiation of scale items. Cronbach's α, broken half reliability, and retest reliability were used to reflect the reliability of the scale. Amos26.0 was used to evaluate the validity of the scale by confirmatory factor analysis and exploratory factor analysis.

### Items analysis

The decision value method and correlation coefficient method [20] are mainly used. (1) Decision value method: The total score was arranged in ascending order, and the first 27% was defined as the low group (recorded as variable 1), the last 27% as the high group (recorded as variable 1), and the middle score segment (recorded as variable 1). Independent sample T-test was used to analyze the differences between the high and low groups and to observe whether the results were statistically significant (*P* < 0.05). (2) Correlation coefficient method: The correlation between each item of the scale and the total score is calculated. The correlation coefficient > 0.40 is excellent, and 0.3 ~ 0.4 is good. However, whether to delete these items with small correlation coefficients needs to be comprehensively considered [21, 22].

### Construct validity

Exploratory factor analysis (EFA) and confirmatory factor analysis (CFA) were used to test the structural validity of the Chinese version of CBSFC-CC. EFA was analyzed by principal component analysis and maximum variance orthogonal rotation. Before the analysis, the suitability of the target scale for factor analysis was determined according to the Kaiser-Mayer-Olkin (KMO) value (> 0.6) and Bartlett's sphericity test (*P* < 0.05). For CFA, it is necessary to evaluate whether the structural model is reasonable by the size of the fitting index. However, due to the many categories of fitting index, not all need attention. In this study, Chi-square/DOF ratio (χ2/df), Comparative Fit Index (CFI), Tuck-Lewis Index (TLI), and Incremental Fit Index (IFI) were observed. Root Mean Square Error of Approximation (RMSEA), Standardized Root Mean Square Residual (SRMR). The values of CFI, TLI, and IFI range from 0 to 1. The larger the value, the better the fitting effect; The lower the RMSEA and SRMR are, the better fitting effect is reflected. It is generally believed that RMSEA ≤ 0.08 indicates a reasonable or good fitting effect, while RMSEA > 0.08 indicates an average or poor fitting effect [23]. Participants were randomly divided into two groups (*N*1 = 319, *N*2 = 208). One for EFA and the other for CFA.

### Convergent validity and discriminant validity

Convergence validity was judged based on the average variance extracted (AVE). The acceptable range of AVE is 0.36 ~ 0.50. Composite Reliabiliy (CR) mainly evaluates the internal consistency of explicit indicators, and its value ranges from 0 to 1. The Fornell-Larcker criterion was used to test the discriminative validity [24]. Discriminant validity was determined by comparing the square roots of the factor’s AVE with the correlation of the specific factor with any of the other factors; if the square root of AVE was more significant than the correlation coefficient, then discriminant validity was accepted.

### Reliability analysis

Cronbach’s α value, split reliability and test–retest reliability were used to evaluate the scale's reliability. Two weeks after completing the first survey, 30 family caregivers from the participants were randomly contacted as retest reliability subjects and completed the questionnaire again independently. The Pearson correlation coefficient was used to evaluate the retest reliability of the scale. Cronbach's α value of > 0.7 [20]and Pearson's r of > 0.8 [25] indicate that the reliability of the scale is acceptable.

### Ethical considerations

This study was approved by the Medical Ethics Committee of Sichuan Provincial People's Hospital(The approval number is 伦审(研)2022年第250号).The participants were informed that they were voluntary to participate in this study and could withdraw at any time during the survey.

## Results

### The characteristic of participants

In this study, a total of 527 family caregivers of children with cancer were enrolled, and 523 valid questionnaires were collected, with an effective recovery rate of 98.75%. Table 1 shows the demographic information of participants. Mothers (78.4%) in their 30 s and 40 s (57.5%) took care of their children with cancer. A quarter of family caregivers have been in care for one to three years, while more than 10% have been in care for more than five years. Most family caregivers are Han Chinese (94.0%), and there are some ethnic minority families (6%) in Sichuan Province due to its location in southwest China and its connection with Yunnan, Guizhou and Tibet. More than half of the family carers were unemployed (62.2%) and had no fixed weekly rest time (72.1%), and most of the family carers had experience in caring for children (60.6%). The age of the children with cancer ranged from 0 to 18 years old, and 45.7% were over 7 to 14 years old. In general, boys (58.1%) were more than girls (41.9%).

**Table 1:** General data of family caregivers of children with cancer (*N* = 315)

Variables	Groups	N	%
Relation	father	54	17.1
	mother	247	78.4
	Grandpa/grandma	10	3.2
	other	4	1.3
Sex	Male	57	18.1
	Female	258	81.9
Age(years)	< 30	51	16.2
	30 ~ 40	181	57.5
	41 ~ 50	68	21.6
	> 50	15	4.8
Nation	The Han nationality	296	94.0
	The Zang nationality	9	2.9
	The Yi nationality	5	1.6
	Qiang ethnic minority	1	0.3
	other	4	1.3
Religion	No	292	92.7
	Yes	23	7.3
Marriage	spinster	2	0.6
	married	291	92.4
	divorce	20	6.3
	Be bereaved of one's spouse	2	0.6
Education	Junior high school and below	111	35.2
	High school or technical secondary school	92	29.2
	Junior college	64	20.3
	Undergraduate course	40	12.7
	Master degree or above	8	2.5
Place of residence	Rural	103	32.7
	Towns and villages	62	19.7
	Urban	150	47.6
Working condition	unemployed	196	62.2
	part-time job	45	14.3
	full-time job	74	23.5
Per capita monthly household income(rmb)	< 3000	123	39
	3001 ~ 5000	130	41.3
	> 5000	62	19.7
Number of children	1	116	36.8
	2	184	58.4
	3 or more	15	4.8
Child care experience	No	124	39.4
	Yes	191	60.6

### Items analysis

**Fig. 1:**
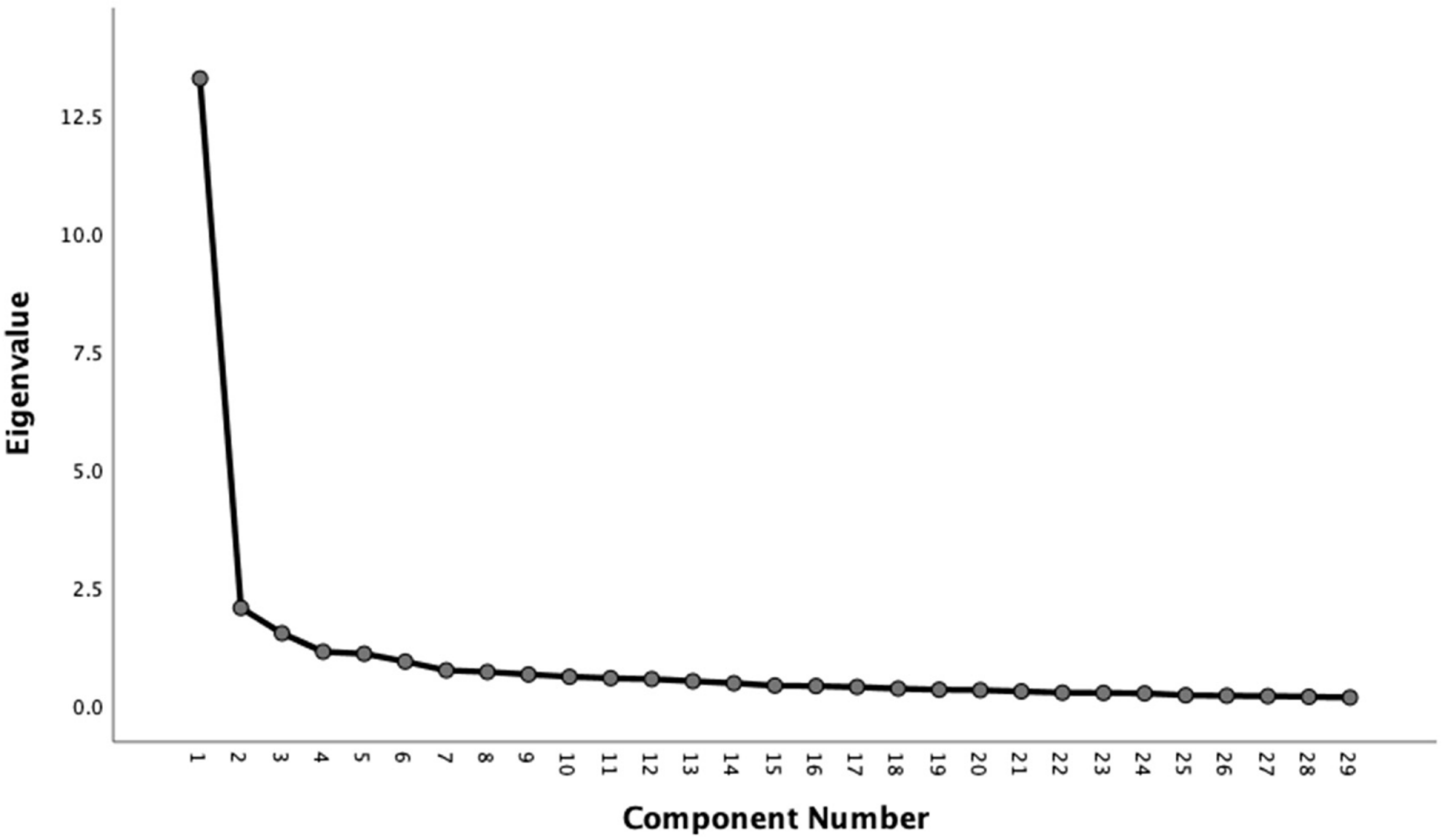
The scree plot

In the study, except item 4, the other items were statistically significant (*P* < 0.01); All items were positively correlated with the total score, but the correlation coefficients of the third and fourth items were less than 0.30, and the correlation coefficients of the other items were 0.33 ~ 0.83, which reached the level above good. The scree plot is shown in Fig. 1. Table 2 shows the results of the items analysis.

**Table 2:** CBSFC-CC item-total score person correlation analysis results (*N* = 315)

Item	Item content	r	*P*
Q1	I suffer from backache	0.54**	< 0.01
Q2	I suffer from headaches	0.65**	< 0.01
Q3	I experience weight gain	0.19**	< 0.05
Q4	I experience weight loss	0.15**	> 0.05
Q5	I suffer from sleep deprivation	0.72**	< 0.01
Q6	I feel exhausted physically	0.69**	< 0.01
Q7	I suffer from pain in my feet	0.55**	< 0.01
Q8	I feel sluggish	0.66**	< 0.01
Q9	I suffer from loss of appetite	0.68**	< 0.01
Q10	I feel guilt	0.64**	< 0.01
Q11	I feel angry	0.66**	< 0.01
Q12	I feel despair	0.74**	< 0.01
Q13	I have pain	0.78**	< 0.01
Q14	I feel a sense of burn out	0.72**	< 0.01
Q15	I feel rage	0.68**	< 0.01
Q16	I feel unhappy	0.70**	< 0.01
Q17	I have difficulty accepting the situation	0.63**	< 0.01
Q18	I feel sad	0.66**	< 0.01
Q19	I feel distant and introverted	0.69**	< 0.01
Q20	I feel inadequate	0.73**	< 0.01
Q21	I have a feeling of loneliness	0.76**	< 0.01
Q22	I am often preoccupied	0.83**	< 0.01
Q23	I experience a lack of focus	0.78**	< 0.01
Q24	I have an adaptation problem	0.73**	< 0.01
Q25	I cannot manage time	0.67**	< 0.01
Q26	I have difficulty deciding	0.70**	< 0.01
Q27	I think my forgetfulness has increased	0.69**	< 0.01
Q28	I experience anxiety	0.77**	< 0.01
Q29	I have a fear of losing my patient	0.65**	< 0.01
Q30	I feel mentally exhausted	0.81**	< 0.01
Q31	I cannot spare time for my self-care (special needs)	0.76**	< 0.01
Q32	I have disruptions in my family order	0.69**	< 0.01
Q33	I experience a change of roles in the family	0.63**	< 0.01
Q34	I cannot spare enough time for fun activities and cultural events	0.58**	< 0.01
Q35	I have communication problems with the people around me	0.61**	< 0.01
Q36	I experience-experienced status loss in my work and social life	0.59**	< 0.01
Q37	I have an increased tendency toward spiritual values and practices	0.33**	< 0.01
Q38	I have financial problems	0.45**	< 0.01
Q39	I feel like my quality of life has decreased	0.63**	< 0.01

^**^Correlation of each item to the total score of the scale. r is the correlation coefficient, P is the probability of observing the test statistic under the assumption that the null hypothesis is true

### Construct validity

#### Exploratory factor analysis

Through component analysis and orthogonal rotation of maximum variance, five common factors with eigenvalues greater than one were extracted for the first time in this study. The variance interpretation rate of the five common factors were 17.62%, 14.17%, 13.68%, 9.16%, and 8.60%, respectively, and the cumulative variance interpretation rate was 63.24% (greater than 50%). Entries 14, 19, 21, 22, 29, 30, 35, and 37 were deleted because of dimensional scrambling and confusion. After deletion, five common factors were extracted again, and the variance interpretation rate was 16.63%, 14.88%, 13.07%, 10.82%, and 10.41%, respectively. The cumulative variance interpretation rate was 65.80% (greater than 50%). However, common factors 3 and 5 contain all items in the dimension of "emotional burden" in the original scale, and the rotational load is similar, so that they can be combined into a common factor. The combined common factor is precisely consistent with the four dimensions in the original scale. The results of the exploratory factor analysis are shown in Table 3.

**Table 3:** Factor load of each item in CBSFC-CC

Item	F1	F2	F3	F4	F5
Q7	0.76	-	-	-	-
Q2	0.75	-	-	-	-
Q9	0.74	-	-	-	-
Q1	0.73	-	-	-	-
Q8	0.72	-	-	-	-
Q6	0.66	-	-	-	-
Q5	0.60	-	-	-	-
Q23	-	0.71	-	-	-
Q25	-	0.69	-	-	-
Q24	-	0.69	-	-	-
Q28	-	0.68	-	-	-
Q26	-	0.61	-	-	-
Q27	-	0.57	-	-	-
Q18	-	-	0.75	-	-
Q17	-	-	0.73	-	-
Q20	-	-	0.56	-	-
Q13	-	-	0.53	-	-
Q10	-	-	0.53	-	-
Q38	-	-	-	0.79	-
Q39	-	-	-	0.76	-
Q36	-	-	-	0.54	-
Q32	-	-	-	0.51	-
Q34	-	-	-	0.48	-
Q33	-	-	-	0.48	-
Q31	-	-	-	0.47	-
Q15	-	-	-	-	0.77
Q11	-	-	-	-	0.69
Q16	-	-	-	-	0.62
Q12	-	-	-	-	0.53

#### Confirmatory factor analysis

**Fig. 2:**
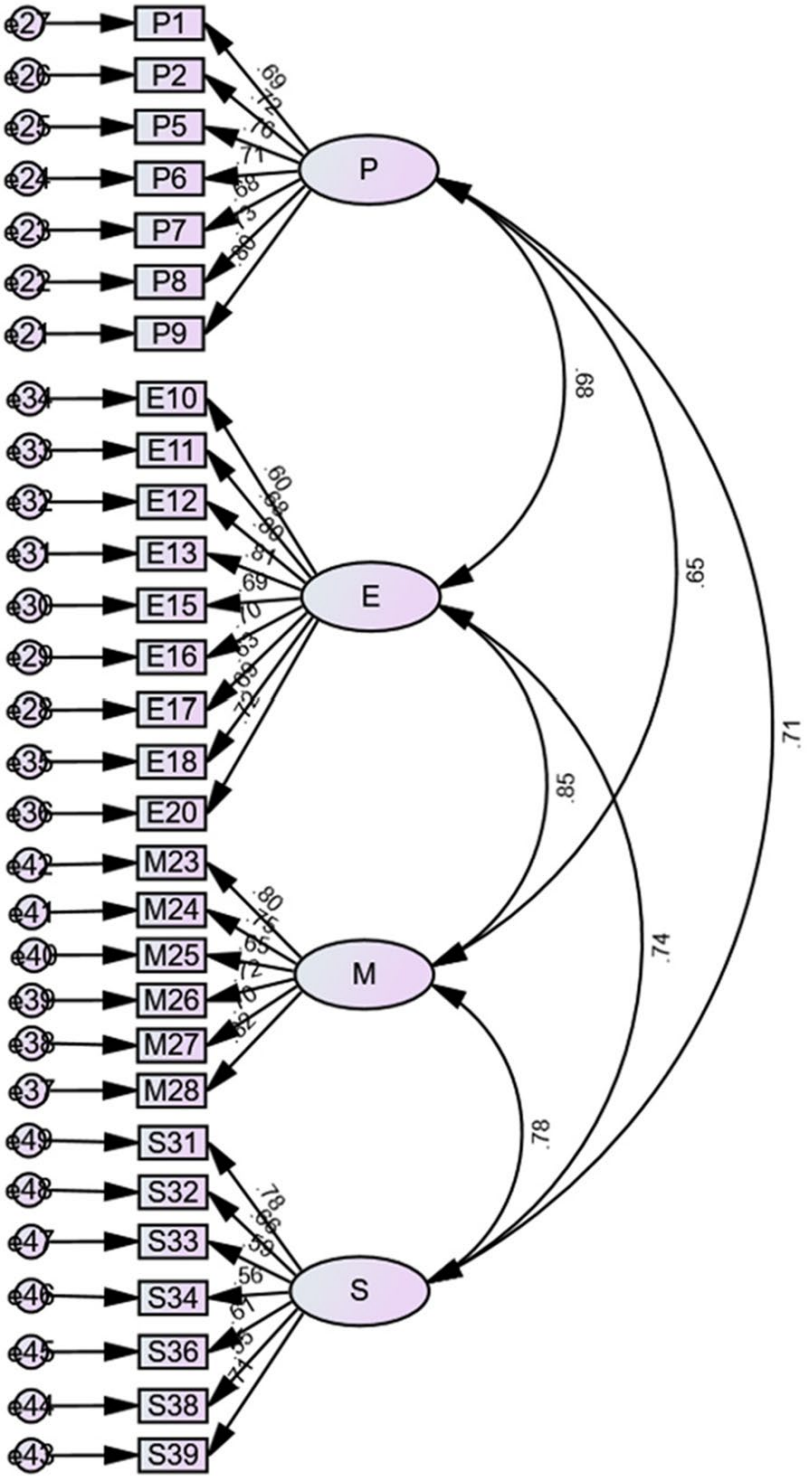
Structure model of the Chinese CBSFC-CC scale

The Chinese version of CBFSC-CC has a total of 4 dimensions, and each dimension has 7, 9, 6, and 7 items. There are 29 entries, namely four potential variables and 29 strength indicators. Latent variables influence each other, each leading indicator influences on the dependent latent variables, and residual measurement exists (see Fig. 2). The correlation fitting coefficients were χ2/df = 2.64, CFI = 0.83, TLI = 0.81, IFI = 0.83, RMSEA = 0.08, SRMR = 0.07. After adding two residual paths according to the correction suggestion, CFI < 0.90 or IFI < 0.90 is found. After discussion with statistical experts, considering the current fitting indicators and the overall situation of the Chinese version of the CBFSC-CC scale, the model is considered reasonable and acceptable.

### Convergent validity and discriminant validity

According to the factor loading results of the Chinese CBSFC-CC scale, a load of all items is greater than 0.5, and *P* < 0.001; AVE values of all dimensions are more significant than 0.40, which is acceptable. CR values were all greater than 0.80. It is shown that the Chinese version of CBSFC-CC has good aggregation validity [26], and convergent validity results are shown in Table 4. The correlation coefficients of each dimension range from 0.364 to 0.553, and the square root of AVE ranges from 0.643 to 0.744, all of which are greater than the correlation coefficients of the corresponding latent variables and explicit indicators, in line with the Fornell-Larcker standard, indicating good discrimination between dimensions. Table 5 shows the discriminative validity results.

**Table 4:** Factor load of convergent validity

Path	Estimated value	AVE	CR
P9 < –- P	0.797***	0.530	0.887
P8 < –- P	0.727***		
P7 < –- P	0.678***		
P6 < –- P	0.712***		
P5 < –- P	0.764***		
P2 < –- P	0.724***		
P1 < –- P	0.686***		
E17 < –- E	0.634***	0.498	0.899
E16 < –- E	0.696***		
E15 < –- E	0.691***		
E13 < –- E	0.812***		
E12 < –- E	0.803***		
E11 < –- E	0.676***		
E10 < –- E	0.602***		
E18 < –- E	0.693***		
E20 < –- E	0.717***		
M28 < –- M	0.825***	0.553	0.88
M27 < –- M	0.699***		
M26 < –- M	0.716***		
M25 < –- M	0.653***		
M24 < –- M	0.752***		
M23 < –- M	0.803***		
S39 < –- S	0.712***	0.414	0.83
S38 < –- S	0.552***		
S36 < –- S	0.612***		
S34 < –- S	0.563***		
S33 < –- S	0.594***		
S32 < –- S	0.66***		
S31 < –- S	0.779***		

^***^Factor load of convergent validity. AVE is average variance extracted, CR is composite reliabiliy

**Table 5:** Discriminative validity results

	P	E	M	S
P	0.53			
E	0.364***	0.498		
M	0.436***	0.469***	0.553	
S	0.431***	0.368***	0.484***	0.414
AVE square root	0.728	0.706	0.744	0.643

^***^Discriminative validity results for each dimension. P is the physical burden, E is the emotional burden, M is the mental burden, S is the sociocultural and economic burden

### Reliability analysis

The Cronbach 'α of Chinese CBSFC-CC is 0.96 for the whole and 0.84 ~ 0.91 for each dimension. The split reliability of Chinese CBFSC-CC is 0.89, and the test–retest reliability is 0.95. Each dimension is 0.85 ~ 0.98. Table 6 shows the results of the reliability analysis.

**Table 6:** Reliability analysis of CBSFC-CC

Variables	Total	P	E	M	S
Cronbach 'α	0.96	0.90	0.91	0.90	0.84
Test–retest reliability	0.95	0.85	0.97	0.98	0.97
Split reliability	0.89				

## Discussion

The number of child cancer survivors is increasing year by year [27], and it is family caregivers who bear the burden of care in their lives. Tens of millions of families will experience or are experiencing the long-term stress caused by cancer [28]. Researchers in the field of pediatric oncology are increasingly conducting research on family caregivers of children with cancer. However, in the earlier research stage of this study, it was found that most domestic and international scholars used the Zarit caregiver burden scale when quantifying the caregiver burden of family caregivers, especially domestic scholars [29–31]. Therefore, this study introduced CBSFC-CC to assess the nursing burden of family caregivers of children with cancer, providing a specific measurement tool for future clinical studies on family caregivers of children with cancer in China.

In this study, the results of the item analysis manifested that all but two items (item three and four) had adequate discriminability for inclusion in this measuring tool. The structural validity of the Chinese version of CBSFC-CC was verified. Structural validity is used to test the degree to which the structure of scale items corresponds to its theoretical concept [32]. Five common factors were extracted for the first time in the exploratory factor analysis results, and the cumulative variance interpretation rate was 63.24%. Due to the phenomenon of dimensional rubbing and double loading in items 14, 19, 21, 22, 29, 30, 35, and 37, we deleted the above eight items based on the psychological measurement characteristics. After removing these items, we reanalyzed the remaining data and extracted the common factors once more. Then five common factors, with a cumulative variance interpretation rate of 65.80%, were extracted, and the dimension rubbing and double loading no longer appeared. In addition, items 14 and 30 are similar in content to retained item 6, both of which aim to assess caregiver fatigue, so they were deleted. Moreover, the deletion of the above items may be closely related to Chinese culture. Compared to Western countries, Chinese culture puts more emphasis on "familism", usually "family-centered decision-making mode". This emphasizes the responsibility of parents in the family [33]. When children are in need, they feel that it is natural for them to be the primary caregivers and will not easily express their negative inner activities to children and the outside world. Instead, they pretend to be in the best possible position to meet the challenge, making items 19, 21, 22, 29, and 35 difficult to measure. At the same time, for Chinese parents, their time is basically taken up by work and family, and few of them care about their spiritual values. For the family of a child with cancer, it is even more difficult to ask the caregiver to pay attention to spiritual life and participate in relevant practical activities. This is because they need to spend more time earning money for the child's medical treatment [34]. On the other hand, it may also be related to the sex ratio and education level of the sample. It has been found that the higher the level of education, the higher the level of self-care, and more likely to pay attention to spiritual needs, and the self-care ability of women may be lower than men [35, 36]. In the sample of this study, however,more than 80% were women, and less than 20% of the sample had a bachelor's degree or higher. Therefore, item 37 is not applicable to family caregivers of children with cancer in China. At the same time, the gravel chart of factor structure was generated. The first four common factors had the largest slope decline in the scree plot. They contributed more to the principal component analysis than the following common factors, indicating that extracting and integrating into four common factors was more reasonable. Therefore, after sorting out the entries of each factor, it was found that the rotation load of common factor 3 (items 10, 13, 17, 18, 20) and 5 (items 11, 12, 15, 16) was similar, and contained all the entries in the dimension of "emotional burden" in the original scale. Based on the entry content and clinical practice [37], it was considered that there was no need to rename the two common factors and can combine them into a common factor. The items contained in other common factors are consistent with the dimensions of the original scale.

In order to further confirm the rationality of this dimension division, this study conducted an in-depth analysis of the dimensions of the scale and the distribution of items contained in it. The results showed that, except for item 4, the other items were statistically significant (*P* < 0.01). According to David, the correlation coefficient method shows that the other items' correlation coefficients are 0.33 ~ 0.83, except for items 3 and 4, reaching a good level or above [37]. To sum up, entries 3 and 4 are considered poor quality and cannot distinguish the caring burden of family caregivers, so they can be deleted, which is consistent with the original scale [17].

In the confirmatory factor analysis, most studies believed that χ2/df ≤ 3, CFI > 0.90, TLI > 0.90, IFI > 0.90, RMSEA < 0.08, and SRMR < 0.08, and the fitting results were good [26], but the critical value has been controversial. The fitting effect will be affected by scale dimension, number of items [38], sample size [39]and other factors. The suggested threshold value is only a reference but not a standard. In this study, the model was modified according to the modification suggestions [40]. Furthermore, paths with residuals of 24 and 25, 37, and 38 were added. However, the modified results could have been better, and the fitting index did not meet the requirements. This situation was discussed with statistical experts. After analysis, statistical experts believed that the overall scale and each dimension had high internal consistency and good stability, which might be affected by scale items and sample size, so this model could be accepted [37]. Adam et al. [40] also believed that when conducting exploratory factor analysis, the limitations of model modification should be acknowledged, and data results should not be excessively relied on. It does not make sense to keep changing the data or adding paths to achieve an ideal fit metric.

Cronbach 's α was used for testing the reliability of the Chinese version of CBSFC-CC. The Cronbach 's α of all dimensions of the Chinese version CBSFC-CC is > 0.8, the half-way reliability coefficient is > 0.8, and the retest reliability coefficient is > 0.9. The Cronbach's α and the retest reliability coefficient of the whole scale are even higher than the original scale [17], and the recommended coefficient at 0.7 [20], indicating that the Chinese version of CBSFC-CC has good reliability and stability, which is consistent with the results of the original study.

### Limitations and implications

There are some limitations in this study and some implications for pediatric nursing clinical work could be proposed. First, the samples in this study are all from Sichuan Province. There are only 5 Grade III and A hospitals in Sichuan Province that carry out pediatric tumor treatment, and the geographical representation is insufficient. Future studies can further expand the source of samples to make the research results more representative. Second, because there is no standard tool to measure the caretaking burden of family caregivers of children with cancer, the validity of the calibration is not discussed in this study. Finally, we hope that the Chinese version of CBSFC-CC can be applied to more studies to explore the current level and influencing factors of caregiver burden of children with cancer in the Chinese context and use this scale to evaluate the effectiveness of relevant interventions.

## Conclusions

Based on the Beaton cross-cultural debugging guide, this study introduced the nursing burden scale for family caregivers of children with cancer abroad. On the basis data of family caregivers of children with cancer in China, the Chinese version of CBFSC-CC was finally formed, including four dimensions (see Appendix). They were physical burden (7 items), emotional burden (9 items), psychological burden (6 items), and social cultural and economic burden (7 items), with a total of 29 items, respectively. It has been verified that the Chinese version of CBFSC-CC has high quality, good reliability and validity, and can effectively measure the level of nursing burden of family caregivers of children with cancer in the context of China.

### Supplementary Information

Supplementary Material 1.

## Data Availability

The datasets generated and/or analyzed during the current study are not publicly available to preserve anonymity of the respondents but are available from the corresponding author on reasonable request.

## Appendix

### Chinese version CBSFC-CC

**Physical Burden**

1. I suffer from back pain;

2. I suffer from headache;

3. I suffer from sleep deprivation;

4. I feel physically exhausted;

5. I suffer from foot pain;

6. I feel sluggish;

7. I suffer from loss of appetite;

**Emotional Burden**

8. I feel guilty;

9. I feel angry;

10. I feel desperate;

11. I feel miserable;

12. I feel angry;

13. I feel unhappy;

14. I can't accept this situation;

15. I feel sad;

16. I feel underpowered;

**Mental burden**

17. I feel inattentive;

18. I have poor adaptability;

19. I cannot manage time;

20. I have a hard time making a decision;

21. I feel more and more forgetful;

22. I am anxious;

**Sociocultural and Economic Burden**

23. I cannot take time off to take care of myself (special needs);

24. My family order was disrupted;

25. My role in the family has changed;

26. I can't spare enough time for fun (social) activities and (important) cultural events;

27. I am experiencing unemployment and feel the decline of social status;

28. I have financial problems (difficulties);

29. I feel that my quality of life has declined.

## Abbreviations

CBSFC-CC: Caregiving burden scale for family caregivers of children with cancer
EFA: Exploratory factor analyses
CFA: Confirmatory factor analyses
ZBI: Zarit Caregiver Burden Interview
CBI: Caregiver burden inventory
CBS: Caregiver burden scale
PECI: Parent experience of child illness
CSI +: Caregiver burden scale (with positive entries)
KMO: Kaiser-Mayer-Olkin
χ2/df: Chi-square/DOF ratio
CFI: Comparative fit index
TLI: Tuck-lewis index
IFI: Incremental fit index
RMSEA: Root mean square error of approximation
SRMR: Standardized root mean square residual
AVE: Average variance extracted
CR: Composite reliabiliy
