# Supplementary material for: Translation and validation of the caregiving burden scale for family caregivers of children with cancer in chinese population
Source: BMC Nurs. 2024 Aug 6;23:534. doi: 10.1186/s12912-024-02204-4 (PMC11302103; doi:10.1186/s12912-024-02204-4)
Supplement: Supplementary file 1 — Supplementary Material 1. [file 12912_2024_2204_MOESM1_ESM.docx]

**Physical Burden**

1. I suffer from back pain；

2. I suffer from headache；

3. I suffer from sleep deprivation；

4. I feel physically exhausted；

5. I suffer from foot pain；

6. I feel sluggish；

7. I suffer from loss of appetite；

**Emotional Burden**

8. I feel guilty;

9. I feel angry;

10. I feel desperate;

11. I feel miserable;

12. I feel angry;

13. I feel unhappy;

14. I can't accept this situation;

15. I feel sad;

16. I feel underpowered;

**Mental burden**

17. I feel inattentive;

18. I have poor adaptability;

19. I cannot manage time;

20. I have a hard time making a decision;

21. I feel more and more forgetful;

22. I am anxious;

**Sociocultural and Economic Burden**

23. I cannot take time off to take care of myself (special needs);

24. My family order was disrupted;

25. My role in the family has changed;

26. I can't spare enough time for fun (social) activities and (important) cultural events；

27. I am experiencing unemployment and feel the decline of social status;

28. I have financial problems (difficulties)；

29. I feel that my quality of life has declined
